# Geriatricians and the older emergency general surgical patient: proactive assessment and patient centred interventions. Salford-POP-GS

**DOI:** 10.1007/s40520-017-0886-5

**Published:** 2018-02-06

**Authors:** Arturo Vilches-Moraga, Jenny Fox

**Affiliations:** 1 Faculty of Medical and Human Sciences, University of Manchester, Manchester, UK; 2 Salford Royal NHS Foundation Trust, Salford, UK

**Keywords:** Emergency general surgery (EGS), Multidisciplinary, Perioperative Care of Older Persons (POPS), Older, Comprehensive Geriatric Assessment (CGA), Frailty

## Abstract

Increasing numbers of older patients require Emergency admission under General Surgery (EGS). This is a group of heterogeneous and often complex individuals with varying degrees of multimorbidity, polypharmacy, functional, mobility and cognitive impairment. Our article describes the benefits of comprehensive assessment coupled with patient-centred multiprofessional interventions and timely discharge planning. We discuss diverse service models and describe our experience in the planning, development and consolidation of a perioperative service for older EGS patients.

## Introduction

The population is ageing. In 2016, 19.2% and 5.4% of the population were aged over 65 and 80 years, respectively [1]. The unstoppable demographic shift, coupled with drastic decreases in elective surgical interventions after the age of 75 years [2], has resulted in higher numbers of older people being admitted as surgical emergencies [3].

Emergency general surgery (EGS) comprises illnesses of very diverse pathology related only by their urgent nature. The most prevalent conditions are hepatopancreaticobiliary and colorectal disorders which together amount to 43% of all EGS diagnoses. The annual case rate (1290 of 100,000) is higher than the sum of all new cancer diagnoses [4].

Although EGS represents 11% of surgical hospitalisations, these are responsible for 50% of all in-hospital morbidity and mortality [5, 6] and account for 50% of a general surgeon’s workload [7]. Patients aged 70 years and older account for 35% of EGS admissions [8] and individuals over the age of 80 years have a 67% increase in the risk-adjusted odds of mortality compared to their younger counterparts [9].

A third of EGS patients undergo a surgical intervention. Older people represent almost half of patients undergoing emergency laparotomy every year in the United Kingdom [10] and 28.8% of those undergoing major emergency general surgery in the United States. An American study of 421,476 patients requiring EGS between 2008 and 2011 [11] showed that seven procedures including partial colectomy, small-bowel resection, cholecystectomy, operative management of peptic ulcer disease, lysis of peritoneal adhesions, appendectomy, and laparotomy collectively accounted for 80% of procedures, 80.3% of deaths, 78.9% of complications, and 80.2% of inpatient costs nationwide. The most common complications after surgery are bleeding (6.2%), incisional surgical site infection (SSI) (3.4%), pneumonia (2.7%), and organ/space SSI (2.6%). Bleeding has the greatest overall impact on mortality (95% CI 8.2, 13.1%, *p* < 0.001) and end-organ dysfunction (95% CI 13.9%, 16.7%, *p* < 0.001) [12]. The development of complications in the postoperative period increases in-hospital mortality significantly (OR 2.51, 95% CI 1.210–5.187, *p* = 0.013) [13].

## Multimorbidity, polypharmacy and frailty

Although over three-quarters of older EGS patients have multimorbidity (coexistence of two or more chronic medical conditions in one individual) and more than half are affected by polypharmacy, neither of these factors influence the uptake of surgery nor worsen hard clinical outcomes such as mortality, length of stay or readmission to hospital [14, 15].

The OPSOC collaboration in the UK found that frailty was present in 28% of 325 EGS patients and established a significant association with increased length of stay and mortality [16]. Similar figures have been described in North America with frailty present in 37% of 220 EGS patients, who were also demonstrated to have an increased risk of complications [17].

Although it is thought that frailty may be modifiable [18], the evidence advocating routine measurement of frailty in older patients remains limited.

## Delivery of care

There are certain interventions proven to be of benefit in managing EGS patients [19, 20]. Amongst these, early review by a consultant surgeon together with urgent imaging of the abdomen reported by a consultant radiologist enabling accurate diagnosis, risk stratification and decision-making. Optimisation of medical problems and pre-operative involvement of a consultant anaesthetist when a surgical intervention is likely to benefit the patient has been shown to improve clinical outcomes. Those patients undergoing major operations and those whose operative mortality risk exceeds 5% should be transferred from theatre into a high dependency area post-surgery.

**Fig. 1:**
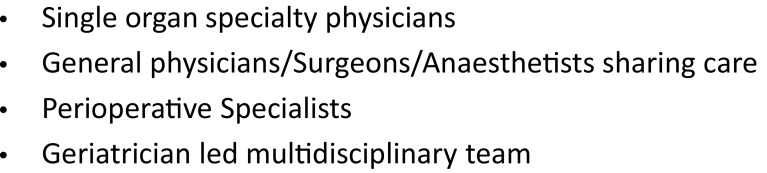
Models of care in emergency general surgery

As shown in Fig. 1, there are a number of models of care aimed at surgical patients requiring medical review [21]. Single organ specialty physicians often provide input to patients admitted to general surgery on an ad hoc basis. This is a reactive service triggered once complications have already developed, and particularly in older surgical patients, it may result in several referrals and even conflicting recommendations.

General physicians, particularly in the United States, advocate a model characterised by joint care of all surgical patients between a physician, anaesthetist and surgeon where the former coordinates the inpatient pathway, whilst the latter provide timely perioperative care [22]. This model appears to work with younger patients; but teams may lack specific skills, training and expertise in the management of complex older people.

In recent years, there has been an upsurge of clinicians with additional subspecialty training in pre-operative assessment, optimisation, and delivery of postoperative care [23]. Whilst adopting this model may be achievable in large NHS Trusts, it may not be feasible in smaller hospitals where team building and consultant retention is challenging. Its expansion will also depend on the establishment of nationwide specialist training programmes.

Older people are a heterogeneous population with a high prevalence of multimorbidity and polypharmacy superimposed on age-related anatomical and physiological changes. This population are at risk of complications such as delirium, functional decline, incontinence, undernutrition and pressure sores [24, 25]. In response, professional bodies and medical societies recognise the importance of Medicine for Care of Older People (MCOP) teams and advocate geriatrician involvement in the care of EGS patients [26]. Comprehensive Geriatric Assessment has become an established multimodal approach to valuation of physical, psychological, functional and social issues in older people [27]. CGA, when coupled with patient-centred multidisciplinary targeted interventions, has been shown to improve outcomes such as length of stay, rates of institutionalisation and readmission, and functional status in oncology [28], vascular [29], orthopaedic [30] and elective general surgery [23]. Unfortunately, only a small proportion of existing geriatric medicine departments (38 of 130 respondents in a 2014 UK study [31]) provide proactive input into EGS patients and only 17% of emergency laparotomy patients over 70 years of age were reviewed by MCOP teams in 2015–16 [10].

## Salford-perioperative care of older people-general surgery (Salford-POPS-GS)

Salford Royal NHS Foundation Trust tailored previous guidance [32–34] to address the needs of older EGS patients. We have developed a general surgical in-reach service in the last 3 years, and in this time have had input into more than 1300 older people. Two consultant geriatricians provide five direct clinical care sessions to the general surgical wards weekly.

Overall, responsibility for patients remains with the parent surgical team but during their hospital stay patients undergo multiprofessional assessment and interventions, regular clinical review by geriatricians, and weekly multidisciplinary team (MDT) discussion.

**Fig. 2:**
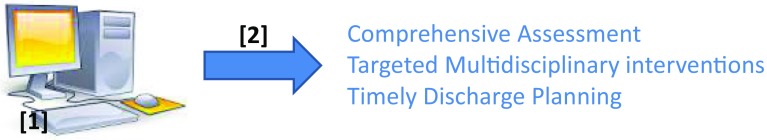
Main components of Salford-POP-GS Surgical In-reach Team. (1) Proactive, daily case finding via electronic patient records of patients over 74 years of age. (2) Multidisciplinary Team work

**Fig. 3:**
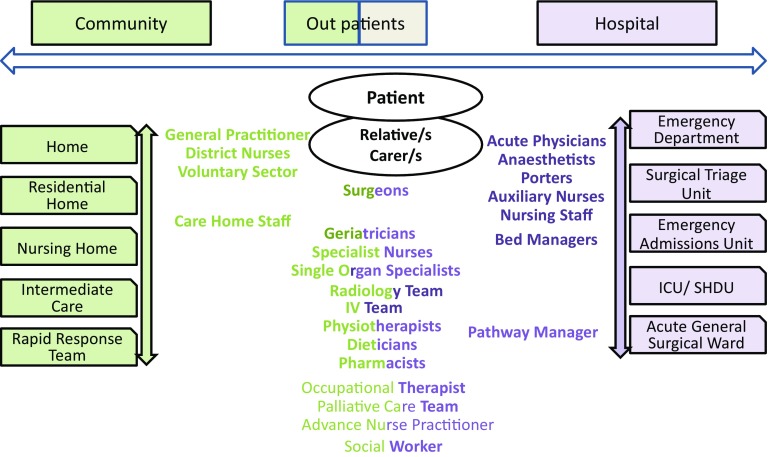
Members of the Surgical Multidisciplinary Team

For each older person, our team proactively scrutinise the electronic patient record before clinical assessment is undertaken. Figure 2 shows a schematic representation of the main components of our service. Figure 3 shows different members of the MDT and interactions between primary and secondary care.

**Fig. 4:**
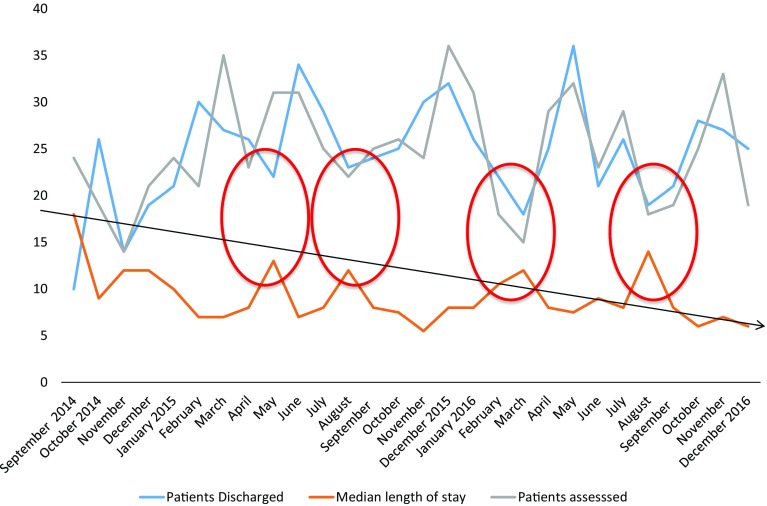
Salford-POPS-GS September 2014 to December 2016. Patients assessed, discharged and their length of stay. Circles represent periods of time with significant reductions in number of patients assessed/discharged and longer LOS

We routinely provide care for patients managed medically or with non-surgical interventions such as therapeutic endoscopy including cholangiopancreatography and stent insertion, and cholecystostomy. We also review the majority of older people managed with a surgical procedure, peri-operatively. Figure 4 demonstrates the number of patients assessed by our service and discharged between September 2014 and December 2016, as well as their median length of stay (LOS). The 1st February 2015, was when our in-reach service was deemed fully consolidated and median LOS was shown to drop from 12.2 days before this date to 9 days after. Our service is reliable except when one of the consultant geriatricians is away on prolonged annual leave. In these circumstances, number of discharges drop and length of stay has been shown to increase as shown in Fig. 4. All four red circles coincide with leave taken by one of the geriatricians.

While a majority of older people are reviewed by our service, pre-operative assessment and optimisation prove challenging given our limited resources. We hope that the inclusion to the team of an Advance Nurse Practitioner will allow us to do more of this work as well as providing better continuity of care, and avoid the demonstrated variations in discharge rate and LOS.

Early CGA delivered by our team has allowed stratification of risk, and supported decision making around suitability for surgery. It has also been shown to minimise adverse drug events and polypharmacy, and allowed early identification and treatment of complications when they arose. Working as a multidisciplinary team has streamlined discharge planning, optimised the use of rehabilitative resources, and improved discussions between health professionals, patients, carers and relatives. The team’s efforts have resulted in reductions in hospital length of stay, lower readmission rates and improvements in coding. The service has also reduced involvement of other specialist teams and urgent reviews by the medical on call team, resulting in better continuity of care. Patient and staff satisfaction have improved.

## Risk stratification

Older people are a heterogeneous population with a spectrum of pre-hospital states varying from the robust to the very dependent [3, 5]. Currently, with improved pharmacotherapy and interventions to manage co-existing multimorbidity, age should no longer be an absolute contraindication to surgery. Indeed, studies that have demonstrated a causal relationship between age and mortality include significant confounding factors as a caveat to interpretation. There is a significant evidence base supporting the use of both P-POSSUM and American Society of Anaesthetists (ASA) score [35, 36] in predicting in-hospital mortality. However, in our experience, impaired cognition and functional status, and the presence of incontinence also reliably predict in-hospital mortality. These are domains that are rarely considered by surgical teams at patient presentation but are routinely assessed as part of CGA.

There is a subset of patients in which active management is considered unsuitable or futile, for example, patients who are in ASA Class V or who present with inoperable disease. In this setting, patients and/or their family and carers should be encouraged to play an active role in decision making and advanced care planning. The geriatrician has a key role in empowering patients and their caregivers to set realistic goals and avoid futile care or unnecessary procedures if they are approaching the end of their life.

## Conundrums in emergency general surgery

There is an insufficient evidence base outlining the best way to assess, manage and rehabilitate older EGS patients and this needs addressing as a matter of urgency.

Randomised controlled and cohort studies are necessary to determine the most efficient models of care and to construct reliable and accurate risk stratification tools when considering emergency surgery. The cost-effectiveness of such services would also need to be determined for sustainability.

## Conclusions and key messages

- The population is ageing and increasing numbers of older people will require EGS.
- While elective surgery rates decrease drastically in patients over the age of 75 years, rates of emergency interventions rise.
- Although older EGS patients form a heterogeneous group of individuals, advanced age, polypharmacy and multimorbidity are the norm.
- Hospital length of stay and mortality, complication, rehospitalisation and institutionalisation rates are significantly higher in older people compared to younger individuals.
- Comprehensive Geriatric Assessment, targeted patient-centred multidisciplinary interventions and timely discharge planning improve clinical outcomes in older EGS patients.
- Anaesthetists, surgeons and geriatricians should receive formal training in the assessment and management of older EGS patients.
- Collaborative care is the future.
- There is an urgent need to carry out high quality research into models of care, risk stratification, pre-operative optimisation and frailty.

We advocate a personalised, multidisciplinary and holistic approach for all older surgical patients, particularly those presenting acutely. Collaborative work involving surgeons, anaesthetists, intensivists, geriatricians and the multidisciplinary team is of paramount importance to optimise clinical outcomes and patient experience.
